# Mr. Alder, on Contagion and Fever

**Published:** 1807-06

**Authors:** Thomas Alder

**Affiliations:** Edinburgh


### *To the Editors of the Medical and Phyſical Journal.*

GENTLEMEN,

IN objecting, as I formerly did, to some expressions used in a joint paper by Dr. Dancer and a Mr. Brown; that is, to the apellations of "fools," and "people who shake all human testimony," and "*court not truth but public fame,*" applied indiscriminately to all those who do not admit fevers are caused by contagion; and in excepting, as I now do, to remarks made by Mr. Brown, in return for what I observed upon that paper, I beg it may not be understood I feel hurt at opposition, or take notice of it altogether on my own account. Much difference surely exists between fair reasoning, however poignant it may be, and mere abuse: in conducting the most arduous and forcible opposition I was capable of, against doctrines which could not but appear to me injurious or absurd, if not highly destructive, I have not made my remarks bear upon a single individual, so as to give him much pain, or do him injury; though I perfectly well knew who those were, who maintained such doctrines, and had not been very handsomely treated by many of them. I could not suppose I alone, or chiefly, was pointed at by Dr. Dancer's letter; but I found the acrimonious remarks contained in it, were applied to *all* who do not see, or think they see, the existence and agency of "febrile contagion," so clearly as the author of them thinks he does; and I had long publicly brought evidence to shew that fevers are caused by bad air; and had more recently adduced many arguments, to shew that no such thing as a general or putrid contagion exists: indeed, I believe I was the first who supported both of these positions. It certainly therefore became me to notice the above-mentioned expression:—but I should not have done this, had I not seen they greatly tended to occasion mischief.

In tracing the history of many, if not most discoveries, we generally find they have been impeded or repressed by blind and malignant opposition; and thence may conclude others have been altogether prevented. And by reflecting on human nature, we find, that though bold, when candid opposition leads to the elucidation and establishment of truth, angry and personal remarks, without reason, lead to quarrels, and to the total extinction of all regards for science.—But after noticing it was not easy for me to see the remarks I complained of, were made by Dr. Dancer, (as they appeared as a postscript to Mr. Brown's letter) I shall leave all reference to any individual, and shall confine myself to those subjects, which originally engaged my attention.

It no doubt will appear very bold, to say the yellow fever is not occasioned by contagion; yet Dr. Miller and Dr. Rush have said this, and their opinion has been received with great respect and complacency by the American Government:—It may seem contemptible and reprehensible, to allege the plague is not caused by contagion; yet Mr. Assalini has lately done this, and nobody at present has invalidated his arguments. Dr. P. Russell has been supposed to have settled the question about the plague being contagious; but any unprejudiced person of judgment, will see he has done no such thing. I beg to represent that the question, "Do fevers arise from contagion?" is one of the most important that ever agitated the public mind. And, that the Laws of Quarantine are better calculated, than all the efforts of Buonaparte, to destroy this nation.

You, gentlemen, will grant we preserve our rank as a nation, merely from the success of our commerce; and that if our commerce fail but a little, our revenue must decrease, and our wealth decline, so that we cannot pay our taxes. But the quarantine laws are so oppressive, that in cases of *general epidemics*, no trade can be carried on.—This is an alarming assertion, but considered as a general one, I am persuaded it will be found to be true; and to be deserving of the greatest attention, even of the legislature. For though France, Spain, Holland, and several other countries, cannot rival us at present, they are likely to undersell us whenever there shall be peace. And from the smallness of their national debt, their revenues can better allow of the clogs their trade must suffer, if these nations enforce quarantine as we do. But America seems very much disposed to disencumber itself of the severity of the quarantine laws; and other nations may follow her example. Yet, allowing all nations are as strict about quarantine as we are, as we have twice as much trade as any other nation, in cases when the quarantine laws are put in force, we must suffer twice as much as any other nation; and from our expenditure exceeding our revenue, we cannot afford to suffer so much as any other nation can.

It may to some seem a matter quite theoretical, to talk about about fevers prevailing so generally, as to injure our commerce to any extent; but a little reading will prove it is not so; in the time of Marc Anthony, there were *two* epidemics, which attacked the whole then known world. Since then epidemics have often been very general; but, suppose they visit only the shores of the Mediteranean and Portugal, or the West Indies, (suppositions which are likely *often* to be realized) they will for a considerable time deprive us of the Levant trade, and the trade of the West Indies. It is true, the plague in the Levant seldom prevails for more than two or three months at a time, but it has continued with more or less violence for two or three years. If we view the quarantine laws in another light, we shall not find their tendency is to promote health or security; for if contagion were aboard any ship, no quarantine could remove it, as the smallest particle of contagion is capable of exciting fever, equally as the greatest quantity. Then, if we turn our thoughts to the circumstances which gave rise to the recent extension of the quarantine laws, we shall find they are some abstract speculations of a few individuals.

Dr. Russell lived when the plague was common, and he recommended greater strictness in the performance of quarantine, and Dr. Hogarth found the air did not communicate contagion beyond a few yards from the place where it arose. It is not difficult to shew Dr. Russell knew no more about contagion than any other person does; and as for Dr. Hogarth, the inference from which he discovered, or pretended to have discovered, is, that the air does not communicate contagion at all. But as it was evident, fevers in many places, and on many occasions, very extensively spread, the doctrine of *fomites* was introduced; thus, it having been found that several substances, such as clothes, furniture, paper, &c. usually passed between febrile patients, and those who were afterwards seized with fever, it was set down, that such substances communicated contagion from the sick to the healthy, and by that means spread the disease.

If you think these cursory preliminary observations worth insertion, I will in my next, adduce arguments to prove that the doctrines which gave rise to the quarantine laws are totally erroneous.

I am, &c.

THOMAS ALDER.

*Edinburgh, March* 6, 1807.

